# Genome-Wide Association Mapping of Root Traits in a Japonica Rice Panel

**DOI:** 10.1371/journal.pone.0078037

**Published:** 2013-11-05

**Authors:** Brigitte Courtois, Alain Audebert, Audrey Dardou, Sandrine Roques, Thaura Ghneim- Herrera, Gaëtan Droc, Julien Frouin, Lauriane Rouan, Eric Gozé, Andrzej Kilian, Nourollah Ahmadi, Michael Dingkuhn

**Affiliations:** 1 Centre de Coopération Internationale en Recherche Agronomique pour le Développement (CIRAD), UMR AGAP, Montpellier, France; 2 Universidad Icesi, Departamento de Ciencias Biológicas, Cali, Colombia; 3 Centre de Coopération Internationale en Recherche Agronomique pour le Développement (CIRAD), UPR SCA, Montpellier, France; 4 Diversity Arrays Technology Pty Ltd. (DArT P/L), Canberra, Australia; United States Department of Agriculture, Agricultural Research Service, United States of America

## Abstract

Rice is a crop prone to drought stress in upland and rainfed lowland ecosystems. A deep root system is recognized as the best drought avoidance mechanism. Genome-wide association mapping offers higher resolution for locating quantitative trait loci (QTLs) than QTL mapping in biparental populations. We performed an association mapping study for root traits using a panel of 167 japonica accessions, mostly of tropical origin. The panel was genotyped at an average density of one marker per 22.5 kb using genotyping by sequencing technology. The linkage disequilibrium in the panel was high (r^2^>0.6, on average, for 20 kb mean distances between markers). The plants were grown in transparent 50 cm × 20 cm × 2 cm Plexiglas nailboard sandwiches filled with 1.5 mm glass beads through which a nutrient solution was circulated. Root system architecture and biomass traits were measured in 30-day-old plants. The panel showed a moderate to high diversity in the various traits, particularly for deep (below 30 cm depth) root mass and the number of deep roots. Association analyses were conducted using a mixed model involving both population structure and kinship to control for false positives. Nineteen associations were significant at P<1e-05, and 78 were significant at P<1e-04. The greatest numbers of significant associations were detected for deep root mass and the number of deep roots, whereas no significant associations were found for total root biomass or deep root proportion. Because several QTLs for different traits were co-localized, 51 unique loci were detected; several co-localized with meta-QTLs for root traits, but none co-localized with rice genes known to be involved in root growth. Several likely candidate genes were found in close proximity to these loci. Additional work is necessary to assess whether these markers are relevant in other backgrounds and whether the genes identified are robust candidates.

## Introduction

Rice (*O. sativa* L.) is the main staple food crop worldwide. In 2011, rice crops occupied 164.1 M ha (http://faostat.fao.org [accessed 28/01/2013]). Rice is grown in a variety of environments, covering a wide range of latitudes and altitudes. This crop exhibits a relatively high water demand in comparison with other cereals, and it is characterized by a broad range of adaptation in terms of the hydrological conditions tolerated. The hydrological conditions of rice ecosystems range from fully aerobic (upland rice) to temporarily (rainfed lowland rice or floating rice) or fully anaerobic (irrigated rice) [1]. Adaptation to a given hydrological regime imposes specific requirements in terms of tolerance to abiotic constraints (submergence or drought). A deep root system, in place before the onset of drought, with thick roots and an extensive branching ability is considered a major component of drought avoidance in rice, enabling the plants to extract water from deep soil layers, provided there is water in the soil profile [2,3]. Rice exhibits a large variability in root traits [4] that is related to both the organization of the species into varietal groups and the adaptation of these groups to a specific ecosystem [5]. As a general trend, indica varieties, adapted to the aquatic ecosystems, tend to have a high number of shallow and thin roots with low root/shoot mass ratio while tropical japonica varieties, grown in the upland ecosystem where the risk of drought is high, have a smaller number of roots, which are deeper and thicker, and a higher root/shoot mass ratio. However, the ability of a variety to develop a deep root system is greatly affected by the physical, chemical and biological conditions of the soil, and sub-optimal conditions can substantially reduce differences between varieties through genotype x environment interactions [6]. 

Plant phenotyping methods are improving rapidly due to the development of high-throughput platforms and image analysis software packages [7,8]. Several platforms specialized on the characterization of the root systems of plants of different ages (from seedling to mature root systems) or in different growth environments (from Petri dish to field) and associated imaging systems have recently been developed [9-12]. However, despite this progress, root traits remain among the most difficult traits to measure and to breed for. Indirect selection systems based on molecular markers linked to root traits appeared early on as a potential way to circumvent this problem. Since the first study by Champoux et al. [13], numerous genetic studies based on QTL detection in biparental rice populations have been conducted. These studies have led to the identification of many QTLs and a few hotspots (reviewed by Courtois et al. [14]). Near isogenic lines have been developed either in the background in which the QTLs were detected or in other backgrounds to validate the phenotypic effects of some of these QTLs [15,16]. These results have led to the first release of a variety with an improved root system obtained by marker-aided selection [17]. The cloning of root QTLs is ongoing. A first gene underlying a QTL for phosphorus uptake, *PSTOL1*, has been identified and appears to be involved in early root growth [18]. A QTL for root angle, *Dro1*, has been cloned [19]. Other research programs are not far behind. However, many of the QTLs that have been identified in mapping populations were not located with sufficient precision to make identifying the underlying gene viable. Whole-genome association mapping offers better resolution and has recently been shown to be effective in reducing the number of candidate genes underlying individual QTLs, notably in rice [20-22]. Linkage disequilibrium decay, which determines the resolution to be expected in the whole-genome association mapping approach, has been reported to range from 500 kb in the temperate japonica rice background to 75 kb in the indica background [20,23]. This range (~ 2 to 0.3 cM) represents a significant improvement in comparison with the confidence interval of QTLs detected by linkage mapping in biparental populations. If the lower estimate of 75 kb is used, approximately 5,000 well-distributed single nucleotide polymorphisms (SNPs) would be needed to scan the whole rice genome of 390 Mb. One of the limitations of the association mapping approach is the high risk of false-positive associations in structured panels [24]. The risk of false positives is particularly high in rice because the genetic structure of *O. sativa* is strongly bipolar, with two major sub-species (indica and japonica) that are thought to have taken different evolutionary paths since their domestication [25] or to originate from two different domestication events [26,27]. Statistical methods enable efficient correction for various levels of population relatedness [28]. However, association mapping reaches its limit when the genetic organization of the panel closely follows the distribution of its phenotypic variability [24]. In such cases, correction for population structure will lead to the elimination of true positives linked to panel structure, creating false negatives. Such a situation is expected for root traits in rice because indica and japonica sub-species have distinct root characteristics [5,29]. One way to avoid this problem is to work with less structured panels, such as panels composed of accessions belonging to just one of the two main rice sub-species, provided that the phenotypic diversity within the sub-panel is sufficient for the trait considered. Tropical japonicas are known to be the best source of deep and thick root varieties, and they also exhibit a large degree of within-group variability. 

The limited polymorphism expected in a panel with a narrow base is not as problematic with the development of new sequencing technologies. The genomes of several rice varieties have been sequenced, and a very large number of SNPs and indels have been found [30]. The SNP frequency is evaluated at 1.0 SNP/130 bp among indica accessions and 1.0 SNP/260 bp among tropical japonica accessions [20,30,31]. Genotyping by sequencing (GBS) methods that combine a reduction of genome complexity using restriction enzymes with sequencing using new sequencing technologies have been shown to efficiently provide the marker density needed for association studies [32,33].

We present here the results of an association mapping study for root depth and associated traits in a panel of japonica accessions genotyped with SNPs derived from GBS.

## Materials and Methods

### Plant material

The panel used in this study was composed of 168 traditional and improved japonica accessions (Table S1). The accessions in the panel were mainly tropical accessions, with a few temperate accessions included for reference purposes. Two additional accessions, IR64, an improved indica variety, and Azucena, a traditional japonica variety, which are known to have contrasting root systems [34], were used as controls. Seeds of the accessions were obtained either from the Centre de Ressources Biologiques Tropicales de Montpellier or from the International Rice Research Institute (IRRI) gene bank (accession numbers in Table S1). For each accession, the seeds were produced by single seed descent over two generations in a Cirad Montpellier greenhouse to ensure that the samples were homogeneous. Seeds of the panel are available for distribution upon request to the first author of this paper as "Orytage japonica panel".

### Genotyping

Genomic DNA was extracted from the leaf tissues of a single plant from each accession using the MATAB method described in Risterucci et al. [35] and then diluted to 100 ng/µl. Genotyping was conducted at Diversity Arrays Technology Pty Ltd. (DArT P/L), Australia, using a method of GBS combining Diversity Arrays Technology (DArT) and a next-generation sequencing technique called DArTseq™. The method involves genome complexity reduction using *Pst*I/*Taq*I restriction enzymes followed by Illumina short-read sequencing. *Pst*I-specific adapters tagged with 96 different barcodes to encode a plate of DNA samples were ligated to the restriction fragments. The resulting products were amplified and checked for quality. The 96 samples were pooled and run in a single lane on an Illumina Hiseq2000 instrument. The *Pst*I adapters included a sequencing primer so that the tags generated were always read from the *Pst*I sites. The resulting sequences were filtered and split into their respective target datasets, and the barcode sequences were trimmed. The sequences were trimmed at 69 bp (5 bp of the restriction site plus 64 bases with a minimum Q score of 10). A proprietary analytical pipeline developed by DArT P/L was used to produce DArT score tables and SNP tables. The remaining 69 bp sequences were aligned to the Os-Nipponbare-Reference-IRGSP-1.0 pseudomolecule assembly [36] using Bowtie v0.12 [37] with a maximum of three mismatches to recover the position of the restriction site for the DArT markers and the position of the polymorphism(s) within the 69 bp sequences for the SNPs. For the DArT markers, the position given is that of the second base of the 6 base *Pst*I restriction site (5'-C|TGCAG-3') because the mutated base is unknown and can be any of the six. The same sequences were then aligned to the pseudomolecules using BLAST (e-value <1.0 e-20) to assess whether additional sequences could be positioned. The sequences that had only one hit on the pseudomolecules or had more than one hit but with a difference of at least 1.0 e-5 between the first and the second hits were retained for further analyses. When the marker position fell within a Michigan State University-annotated gene (http://rice.plantbiology.msu.edu/), the feature was determined (intron, exon, 3' or 5' UTR), and the name and function of the gene were retrieved. Call rates were measured for all markers, and markers with call rates below 80% were discarded. The allele frequency of the remaining markers was then calculated, and markers for which the minor allele had a frequency below 2.5% were also discarded. 

### Imputation of missing data

The power to detect significant association is linked to population size. To prevent the loss of detection power, missing data were estimated using Beagle v3.3, which enables the inference of haplotypes and imputation of sporadic missing data in large-scale phase-known or phase-unknown genotype datasets [38]. Beagle uses a localized haplotype cluster model. It is a special class of directed acyclic graph which empirically models haplotype frequencies on a local scale and therefore adapts to local structure in the data. The model determines a hidden Markov model that can be used to find the most likely haplotype pair for each individual, given the genotype data for that individual and the graphical haplotype frequency model. The method works iteratively using an expectation –maximization type approach. The imputed missing data, probabilities of missing genotypes and inferred haplotypes are calculated from the model that is fitted at the final iteration. The SNP x accession matrix after imputation is available for download at http://tropgenedb.cirad.fr/tropgene/JSP/interface.jsp?module=RICE as "Orytage dataset".

### Linkage disequilibrium

To evaluate the resolution to be expected in association mapping, the linkage disequilibrium within the panel was evaluated by computing the r^2^ values between pairs of SNP markers in a sliding window of 50 markers using Tassel [39] and tabulating the average r^2^ as a function of the physical distances between markers. A power-law (y=ax^k^) was fitted to the data to determine the physical position (x) corresponding to a given r^2^ value (y). To prevent bias associated with the poor performances of LD indices for markers with very low allelic frequencies [40], only markers with a minor allele frequency greater than 10% were used in these computations.

### Phenotyping

**Figure 1:**
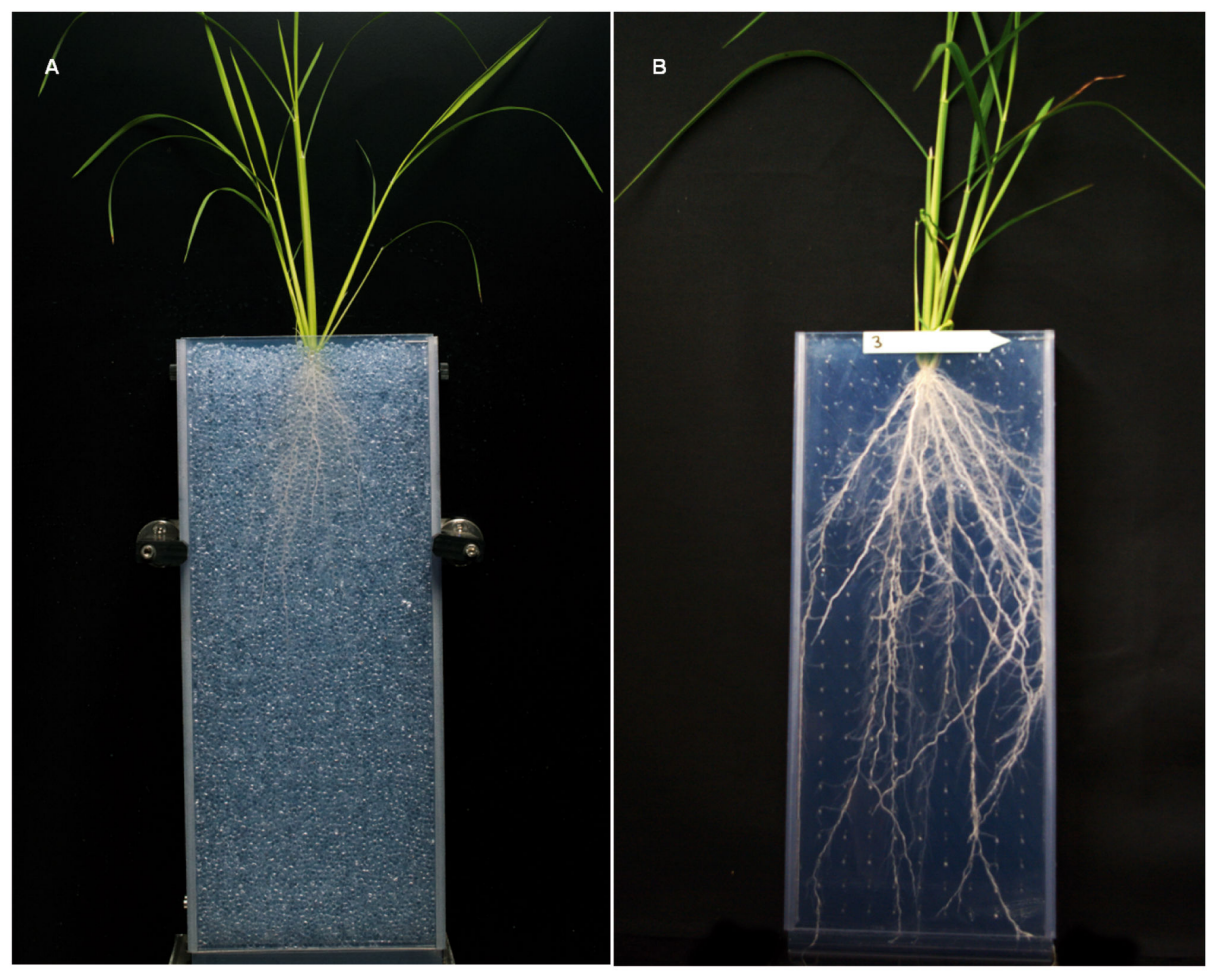
Rhizoboxes used in the Rhizoscope phenotyping platform. a. With beads. b. After bead removal.

**Figure 2:**
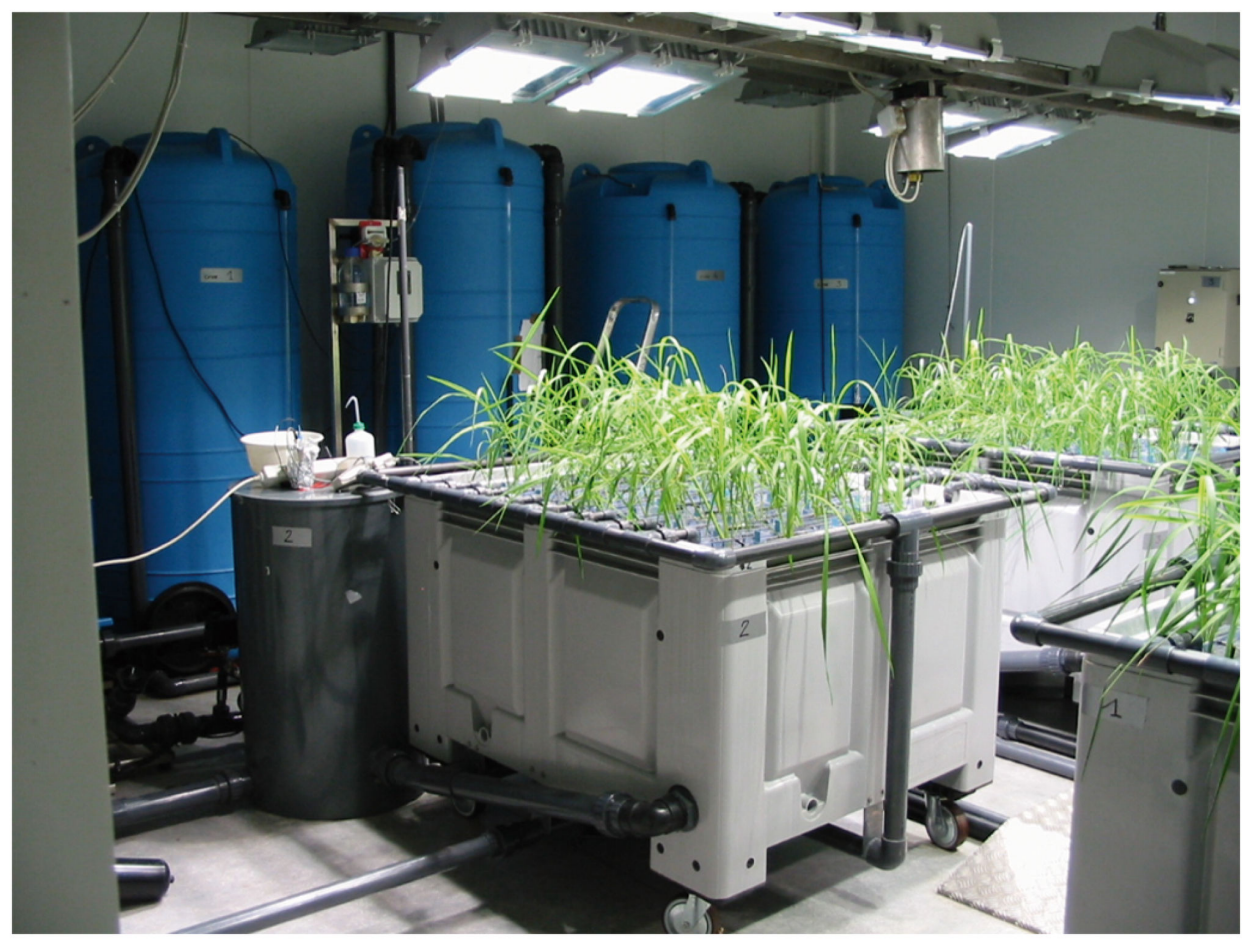
General view of the Rhizoscope phenotyping system.

The plants were grown in a hydroponic system set in a growth chamber developed by Cirad and called Rhizoscope that has the capacity to handle 192 plants at a time [41]. The experimental unit was a sandwich of two 50 cm × 20 cm × 2 cm Plexiglas plates (internal dimensions) filled with glass beads of 1.5 mm diameter, called a rhizobox (Figure 1). A trap at the bottom of the sandwich enabled the easy removal of the beads at the end of the experiment. This device greatly simplifies the cleaning step while imposing some degree of mechanical resistance to root penetration that is closer to normal soil conditions than is a pure hydroponics system. The rhizobox can be completely opened as well. Similar to nailboard systems, each rhizobox contains a grid of nails, which holds the root system in place after bead removal when the sandwich is opened. The 192 rhizoboxes were set in four large tanks with a capacity of 48 rhizoboxes each (Figure 2). An aerated nutrient solution (volume of 3,000 l) was circulated continuously through the rhizoboxes (composition in Table S2). After pre-germinating several seeds per accession at 28°C for three days, one well developed seedling per rhizobox was set on the top of the beads. The solution pH was adjusted to and maintained at 5.4±0.2 by automatic pH controllers. A cooling system maintained the temperature of the solution at 27±1°C. The conditions in the growth chamber were 28°C during the day and 25°C at night with a 12:12 photoperiod. The radiation was 400 to 450 µmol photons per m^2^ per s (PAR). The relative humidity was set to 55%. 

After 30 days of growth (corresponding to a thermal time of 790°C days ), the rhizoboxes were taken out of the tanks, and the beads were removed. The whole root system, which remained in position on the nail plate, was photographed. The angles of the most external left and right crown roots to the vertical were measured with Image J [42] before the roots reached the rhizobox sides and could change direction. The sum of these two angles was used as the angle of the root cone (ANGLE) in subsequent analyses. The number of tillers per plant (NBT) was counted, and the length of the longest leaf (LLGTH) was measured as a proxy for plant height. The deepest point reached by the roots was measured in position (DEPTH) and again after the plants were removed from the rhizobox (LENGTH). The number of crown roots reaching 30 cm depth (NBR_30) was counted. Then, the root system was cut into three segments (0-20 cm, 20-30 cm and below 30 cm). Each root segment was carefully washed to remove the remaining beads, if any, dried in an oven at 72°C for three days and weighed (RB0020, RB2030 and RBB30). The total root biomass (RB) and the deep root biomass (DRB) were computed as the sum of the root mass in all three segments and in the two deepest segments, respectively. Deep root proportion (DRP) was computed as the ratio of DRB*100/RB. In rice, root emission is synchronized with tiller emission according to the phyllochron model [43]. Allometric ratios such as the root-to-shoot mass ratio (R/S) are used to describe the coordination between the growth and development of the roots and shoots [4]. Shoot tissues were similarly dried and weighed (SB), and the plant biomass (PB) and R/S were computed. The root and shoot traits measured are summarized in Table 1.

**Table 1:** List of the measured traits with their abbreviations.

Trait	Abbreviation
Longest leaf length	LLGTH
Number of tillers	NBT
Shoot biomass	SB
Deepest point reached by the roots in position in the plates	DEPTH
Maximum root length	LENGTH
Angle to the vertical of the root cone	ANGLE
Number of roots reaching 30 cm depth	NBR_30
Root dry mass in the 00-20 cm layer	RB0020
Root dry mass in the 20-30 cm layer	RB2030
Root dry mass below 30 cm	RBB30
Deep root biomass	DRB
Deep root proportion	DRP
Root biomass	RB
Plant biomass	PB
Root to shoot ratio	R_S

### Experimental design

The experimental unit was one rhizobox. The experimental design was an alpha lattice with two replicates of 192 rhizoboxes. The two replicates were grown at a three-month interval due to space constraints. In each replicate, the four tanks were considered the main blocks and virtually divided into three sub-blocks of 16 rhizoboxes each. These replicates, blocks and sub-blocks were used as controlled factors for the design optimization and randomization. The two controls, IR64 and Azucena, were included in each sub-block to ensure an additional control for spatial variability. 

### Statistical analysis of phenotypic data

An analysis of variance was conducted considering all genotype and block effects as fixed. These effects were tested using SAS v9.2 (SAS Institute, Cary NC, USA), and the means were adjusted for block and sub-block factors. The adjusted means of all accessions are available for download at http://tropgenedb.cirad.fr/tropgene/JSP/interface.jsp?module=RICE as "Orytage dataset". Broad-sense heritabilities based on genotypic means (h^2^) were computed from the genotype F value of the variance analysis as (F-1)/F [44]. An ANOVA was conducted on the adjusted means to assess the phenotypic differences among sub-populations. Phenotypic correlations were computed from the adjusted means using SAS. Principal component analyses (PCA) were run using some or all of the measured traits with XLStat [45]. The coordinates of the accessions on the main axis together with the adjusted means were used in association mapping.

### Analysis of population structure

The structure of the panel was analyzed using a model-based approach complemented by a discriminant analysis of principal components (DAPC [46]), using a subset of 200 SNP markers that were well distributed in the genome and had no missing data before imputation. The DAPC was used to help determine the most likely number of sub-populations in the panel, which can be difficult with the model-based approach. For the model-based approach, the analyses were conducted with the software Structure [47] with the following parameters: K, the number of sub-populations in the panel varying from 1 to 15; 10 runs per K value; for each run, 200,000 burn-ins and 200,000 iterations; haploid data with the possibility of admixture; and correlated allelic frequencies. The analyses were run on Bioportal (http://www.bioportal.uio.no/). The R Adegenet package [48] was used for DAPC. To illustrate the panel organization, an unweighted neighbor-joining (NJ) tree was constructed based on a dissimilarity matrix computed using a shared allele index with DARwin software [49]; subpopulation attributions derived from the model-based approach were projected on the tree. An accession was discretely assigned to a subpopulation when more than 80% of its genome composition came from that subpopulation. The percentages of admixtures from Structure results (Q matrix) were used as covariates in the models to correct the association tests for false positives. 

### Kinship coefficient

The control of spurious associations is improved when finer levels of relatedness are taken into account by fitting a marker-based kinship matrix in the models [28]. Such control is particularly important for panels involving breeding lines. A simple genetic similarity matrix was shown to work as well as a matrix based on identity by descent for this purpose [50,51]. The coefficients of kinship between pairs of accessions were determined using a set of 2600 SNPs without any missing data. A pairwise dissimilarity matrix was computed based on simple matching index using DARwin [49] and then converted to a similarity matrix (K matrix). 

### Association mapping

Using the adjusted means for observations on each accession, we compared three models for their capacity to fit the data: a General Linear Model (GLM) using the percentages of admixture (Q matrix) as fixed effects, a Mixed Linear Model (MLM) using the kinship matrix (K) as a random effect (MLM1) and an MLM using both Q and K (MLM2). The best model was chosen on a trait-by-trait basis by comparing the likelihoods of each model using the Bayesian Information Criterion (BIC [52]). The BIC was computed as -2 ln(L) + kln(n) where ln is the natural logarithm, L is the maximized value of the likelihood function for the estimate model, k is the number of estimated parameters and n is the sample size. The model with the smallest BIC was selected. Analyses for model comparisons were conducted using either R [53] for GLM, or Tassel for MLM1 and MLM2. Once the model was chosen, the analyses were conducted using the Linux version of FaST-LMM (Factored Spectrally Transformed Linear Mixed Models) that uses an exact method [54]. In an exact method, the additive genetic and residual variance components (i.e., the random effects of the mixed model) are re-estimated for each SNP in a model including the marker effect rather than estimated under the null hypothesis. This approach increases the detection power. For each SNP, FaST-LMM computed a *p-value*, a *q-value* corresponding to the False Discovery Rate (FDR [55]), the log likelihoods of the null and alternative models and the fixed-effect weight of the SNP with its standard error. The threshold to declare a significant association was set at a probability level of 1.0 e-04.

### QTL map

A database of QTLs for root traits had been established previously [14]. The physical position and confidence intervals of 137 QTLs for LENGTH, DRB, RB and R_S extracted from this database were used to build a QTL map using the Spidermap Excel macro ( http://jframi.free.fr/wordpress/). The type of mapping population (japonica x japonica, indica x indica, or other type) in which the QTLs were detected was also recovered from the database. The positions of markers that were significantly associated with root phenotypes in this study, as well as the positions of genes known to be involved in root development or nutrient uptake in rice in the literature ("EURoot genes" set extracted from TropgeneDB: http://tropgenedb.cirad.fr/tropgene/JSP/interface.jsp?module=RICE ), were added to the map to assess co-localization.

## Results

### Marker distribution

The GBS method used yielded 16,664 markers (9,727 DArTs and 6,717 SNPs). Approximately 46% of the markers were in genic regions (5' UTR, exon, intron or 3'UTR), which confirmed that *Pst*I, a methylation-sensitive restriction enzyme, cut preferentially in gene-rich regions. The average heterozygosity, calculated from the SNP markers, was low (1.3%), as expected for DNA extracted from single plants that had been self-fertilized for two generations. The rate of missing data before imputation was 3.8%. Even though the markers with a minor allele frequency below 2.5% had been discarded, the minor allele frequency distribution was still skewed toward low frequencies with an average at 15.5% and 46.2% of the markers with a minor allele frequency below 10%. 

The number of markers corresponded to an average density of one marker per 22.5 kb. The markers were relatively evenly distributed, with 69% of the intervals between markers of less than 20 kb and 96% of less than 100 kb. Only 19 segments of more than 500 kb without markers were found, including two intervals of approximately 1.0 Mb on chromosomes 4 and 5. In addition, long segments with low polymorphism (low marker density with a high proportion of markers with low minor allele frequency) were detected on chromosomes 4 (approximate position 22.0 to 28.0 Mb), 5 (7.5 to 12.5 Mb), 9 (15.0 to 20.0 Mb), 11 (8.0 to 15.0 Mb) and 12 (0.0 to 2.0 Mb) (Figure S1). 

The decay of LD along physical distance is shown in Table S3. For between-marker distances of 0 to 20 kb, the r^2^ value attained 0.66. The r^2^ value decreased to half this initial level at approximately 150 kb between markers and was 0.2 and 0.1 at 475 kb and 2.4 Mb between markers, respectively. A similar trend was observed for all chromosomes, with similar starting values for the 0-20 kb interval, although the LD decay was more rapid on some chromosomes (e.g., chromosome 5) than on others (e.g., chromosomes 3, 4 and 12). These values are consistent with those expected in such a genetic background. They showed that, on average, the LD in the panel was high and did not decrease rapidly with physical distance. The average marker density (one marker per 22.5 kb) was therefore sufficient for whole-genome association mapping. However, the expected resolution, although better than that achievable with a biparental mapping population of the same size, was still far from the gene level which would require a density higher than one marker per 5 kb.

### Panel structure and kinship

Among the 170 accessions phenotyped, two were classified as indica based on their marker patterns. These two accessions and the indica control IR64 were excluded from the association analyses that were conducted on the 167 japonica accessions. The Structure software results suggested that the japonica panel was composed of six subpopulations and a large number of admixed accessions. The subpopulation assignments of the accessions are given in Table S1. The DAPC-based method yielded the same number of subpopulations and the same subpopulation attributions but distributed the admixed accessions into the various subpopulations (data not shown). The structure was partly correlated with geography and partly determined by the breeding program origin. Subpopulation 1, the largest subpopulation (46 acc.), was composed of traditional and improved upland rice varieties from Africa and Latin America, reflecting the intensity of exchanges between the breeding programs in these areas. Subpopulation 2 (30 acc.) was composed mainly of traditional upland varieties of equatorial Asia (Indonesia, the Philippines or Malaysia). Subpopulation 3 (20 acc.) was composed of traditional upland rice varieties from Southeast Asia. Subpopulation 4 (10 acc.) contained several varieties from temperate origins or that had adapted to cold climates. Subpopulation 5 (8 acc.) consisted of Indonesian varieties, some of which belonged to the bulu ecotype, which is adapted to the lowland ecosystem. Subpopulation 6 (6 acc.) was composed of improved accessions derived from the variety Colombia 1. Forty-eight varieties appeared to be admixed. This relatively high admixture percentage (1/3) confirms that gene exchange has occurred among these subpopulations. The projection of the subpopulations on an NJ tree is shown in Figure S2. The kinship matrix recorded values ranging from 0.61 to 0.98, showing a broad range of familial relatedness between pair of accessions.

### Phenotyping root architecture

The analysis of variance (Table 2) enabled us to assess the extent of the experimental noise in our phenotyping system. In most cases, the replicate and block effects were highly significant, whereas the sub-block effect was not. These results indicated some degree of heterogeneity in temperature and humidity in the growth chamber. The genotype effect, involving all accessions except the controls, was highly significant for all root and shoot traits. 

**Table 2:** P values of F-tests following analysis of variance for the different traits.

	Source of variation (fixed effects)			
Trait	Rep	Block(Rep)	Sb(block*rep)	Genotype
LLGTH	<0.0001	<0.0001	0.9824	<0.0001
NBT	0.0374	<0.0001	0.6815	<0.0001
SB	0.0002	<0.0001	0.0175	<0.0001
LENGTH	0.0104	<0.0001	0.2227	<0.0001
DEPTH	0.1666	<0.0001	0.8545	<0.0001
ANGLE	0.0096	0.2403	0.0296	<0.0001
NBR_30	<0.0001	0.0001	0.1650	<0.0001
RB0020	0.0131	<0.0001	0.0157	<0.0001
RB2030	0.6961	<0.0001	0.0230	<0.0001
RBB30	0.4830	<0.0001	0.3319	<0.0001
DRB	0.8820	<0.0001	0.0850	<0.0001
DRP	0.0204	<0.0001	0.2968	<0.0001
RB	0.0491	<0.0001	0.0088	<0.0001
PB	0.0004	<0.0001	0.0090	<0.0001
R_S	0.0005	0.2870	0.9441	<0.0001

Rep = replicate; Block(Rep) = block within replicate; Sb(block*rep) = sub-block within block*replicate.

**Figure 3:**
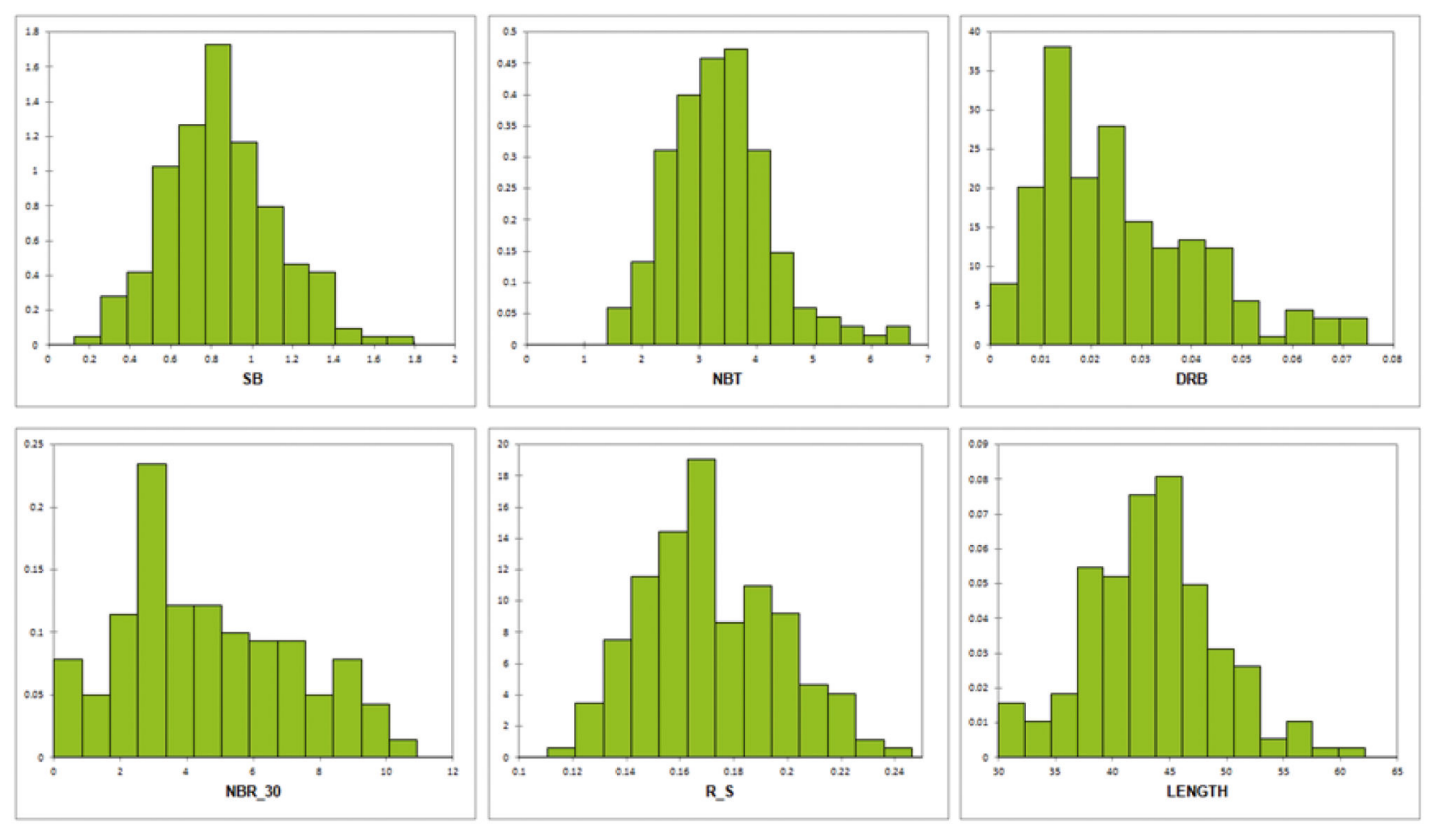
Distribution of selected traits. SB =shoot biomass; NBT = Tiller number; DRB = deep root biomass; NBR_30 = number of roots below 30 cm; R_S = root/shoot mass ratio; LENGTH = maximum root length .

Moderate to large variation was observed for most root parameters, with CVs varying from 13% for DEPTH to 103% for RBB30 (Table 3). The distribution of the root parameters were globally normal, with the exceptions of NBR_30, RB2030 and RBB30, which had skewed distributions due to the presence of accessions with shallow roots that did not exceed 30 cm in length (Figure 3). The broad-sense heritabilities based on genotypic means, which measure the reproducibility of the experiment, were also reasonably high, varying from 0.66 for NBR_30 to 0.89 for DRP. The six subpopulations of the panel differed in terms of means for all traits except for RB0020 (Table 4). Subpopulations 1 (upland rice varieties from Africa and Latin America), 2 (traditional upland varieties of equatorial Asia) and 3 (upland rice varieties from Southeast Asia) were showing the deepest roots (high NBR_30, DRB, DRP, DEPTH and LENGTH) with subpopulation 3 characterized by a larger biomass (NBT, SB, RB and PB) than subpopulations 1 and 2. Subpopulations 4 (temperate accessions) and 5 (bulu types) were showing a large shoot biomass, similar root mass in the shallow horizon than the other subpopulations but much more limited root mass in depth and low R_S. Sub-population 6 (accessions derived from Colombia 1) was composed of small size accessions with limited shoot biomass (low LLGTH, NBT, SB and PB), limited root development and intermediate R_S. The admixed group was intermediate for most traits. 

**Table 3:** Statistical parameters of the association panel for the measured traits.

Trait	N	Minimum	Maximum	Mean	Stdev	CV	Normality	h2
LLGTH (cm)	167	43.096	77.124	58.201	7.227	12.4	Yes	0.84
NBT	167	1.470	6.576	3.363	0.915	27.1	No	0.51
SB (g)	167	0.252	1.693	0.847	0.275	32.4	Yes	0.80
LENGTH (cm)	167	27.774	59.308	41.074	5.447	13.2	Yes	0.84
DEPTH (cm)	167	30.613	61.146	43.657	5.770	13.2	Yes	0.81
ANGLE (°)	167	42.402	92.763	69.753	10.604	15.2	Yes	0.75
NBR_30	167	-0.466	10.773	4.604	2.632	57.0	No	0.66
RB0020 (g)	167	0.042	0.241	0.118	0.037	31.5	Yes	0.79
RB2030 (g)	167	0.001	0.050	0.019	0.010	52.1	No	0.83
RBB30 (g)	167	0.000	0.029	0.006	0.007	103.2	No	0.84
DRB (g)	167	0.000	0.074	0.026	0.016	62.9	No	0.84
DRP	167	2.540	31.714	16.808	6.611	39.2	Yes	0.89
RB (g)	167	0.042	0.296	0.144	0.049	33.9	Yes	0.79
PB (g)	167	0.299	1.945	0.992	0.319	32.1	Yes	0.80
R_S	167	0.098	0.236	0.171	0.026	15.1	Yes	0.89

N = number of observations (indica accessions excluded); Stdev = standard deviation; CV = coefficient of variation of the panel; h2 = broad-sense heritability at the genotype mean level.

**Table 4:** Mean comparisons among sub-populations detected in the panel.

Sub-population	1	2	3	4	5	6	Admixed
LLGTH	55.6 bc	59.9 ab	63.9 a	64.4 a	59.5 ab	52.3 c	56.3 bc
NBT	3.41 ab	2.67 b	3.72 a	4.01 a	4.11 a	3.22 ab	3.35 ab
SB	0.8852 b	0.7355 b	0.9403 b	0.9591 b	1.1851 a	0.7023 b	0.7761 b
LENGTH	44.97 a	46.26 a	46.25 a	38.30 b	42.98 ab	39.00 b	41.39 ab
DEPTH	42.40 ab	43.45 a	43.30 a	36.24 c	39.41 abc	37.28 bc	39.07 abc
ANGLE	71.18 b	68.82 bc	74.04 b	66.21 bc	84.55 a	59.70 c	66.50 bc
NBR_30	5.23 a	5.36 a	5.97 a	2.44 b	2.76 b	2.57 b	3.94 ab
RB0020	0.1177 a	0.1059 a	0.1426 a	0.1425 a	0.1074 a	0.1099 a	0.1074 a
RB2030	0.0212 b	0.0204 b	0.0301 a	0.0118 b	0.0149 b	0.0138 b	0.0157 b
RBB30	0.0079 ab	0.0080 ab	0.0120 a	0.0018 b	0.0029 b	0.0024 b	0.0038 b
DRB	0.0291 ab	0.0284 ab	0.0421 a	0.0136 b	0.0178 b	0.0161 b	0.0195 b
DRP	19.03 ab	18.90 ab	21.60 a	9.46 c	11.30 c	12.03 c	14.59 bc
RB	0.1469 ab	0.1356 ab	0.1846 a	0.1562 ab	0.1556 ab	0.1264 b	0.127 b
PB	1.0318 ab	0.8783 b	1.1239 ab	1.1154 ab	1.3403 a	0.8287 b	0.9031 b
R_S	0.1680 b	0.1811 ab	0.1958 a	0.1657 b	0.1299 c	0.1808 ab	0.1654 b

Within a row, means followed by the same letters are not significantly different at P=0.05.

**Figure 4:**
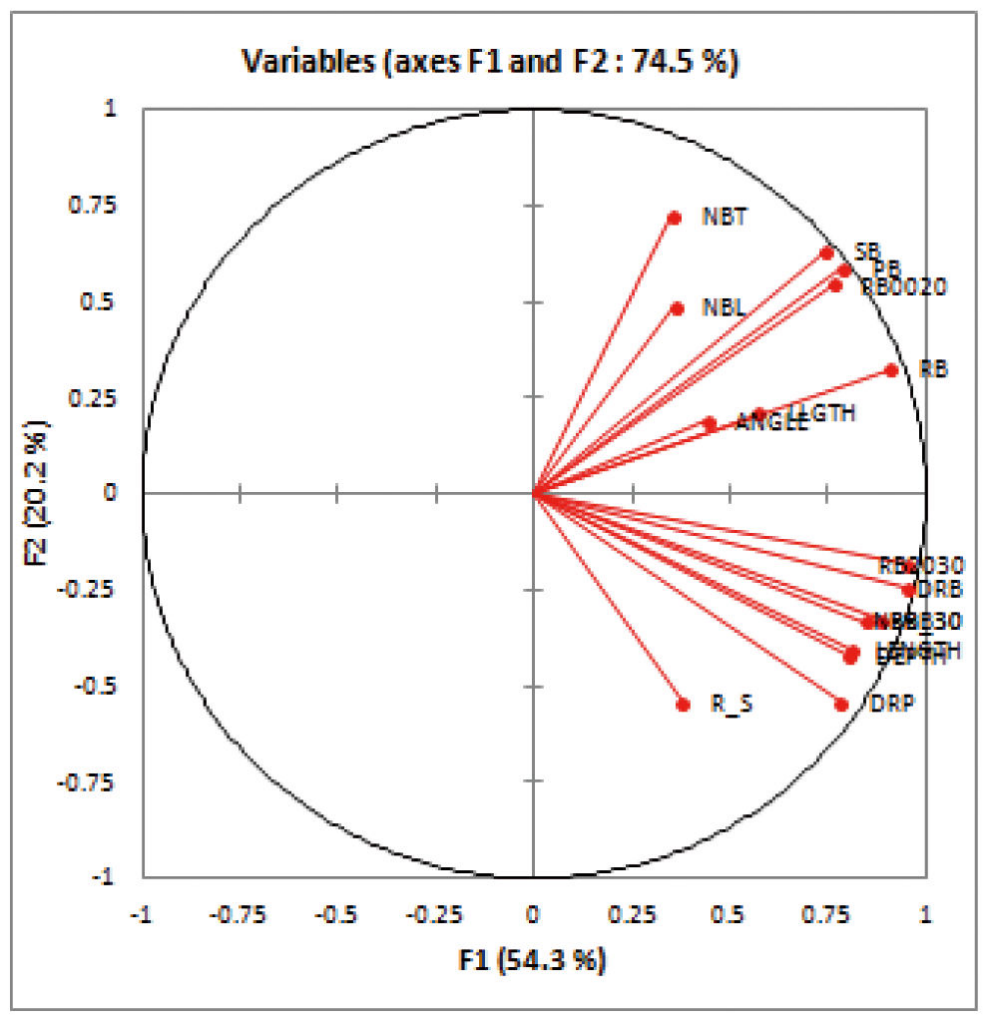
Circle of correlations from the principal component analysis of all traits. LLGTH = leaf length; NBT = tiller number, SB = shoot biomass; DEPTH = maximum depth reached by the roots in position; LENGTH = maximum root length; ANGLE = root cone angle; NBR_30 = number of roots below 30 cm; RB0020 = root mass in the 0-20 cm layer; RB2030 = root mass in the 20-30 cm layer; RBB30 = root mass below 30 cm; DRB = deep root biomass; DRP = deep root proportion; RB = root biomass; PB = plant biomass; R_S = root to shoot mass ratio .

A large degree of positive phenotypic correlations was observed between the traits measured (Table S4). Some of these correlations were expected from the physiological relatedness of the traits. For instance, this was the case for all traits linked with root depth (LENGTH, DEPTH, NBR_30, RN2030 and RBB30) which had correlation coefficients above 0.75 among each other (P<0.0001). However, the root cone angle, which is often considered as a proxy for root depth, showed a weak correlation with root depth in our system and in a direction opposite to what was expected (r^2^ = 0.29 with DEPTH and 0.33 with LENGTH). In the multivariate analysis (PCA) conducted on all the measured traits, the first two principal components summarized 74.5% of the variability. The correlation circle (Figure 4) showed that the first axis was mainly a vigor axis, separating plants with small and large biomasses, whereas the second axis divided shoot or shallow root traits (NBT, LLGTH, SB, RB0020) and deep root traits (LENGTH, DEPTH, NBR_30, DRB or R/S). The accessions on the first plane tended to be grouped by subpopulation (data not shown). A second PCA was conducted using only deep root traits (LENGTH, DEPTH, NBR_30, RBB30, DRB and DRP). The first axis summarized 87.9% of the variation, and the second explained only 5.0% (data not shown). The scores of the accessions on the first axis of the second PCA (PCA1) that separated shallow rooted and deep-rooted accessions were included among the phenotypic traits used in association mapping.

### Association mapping

**Figure 5:**
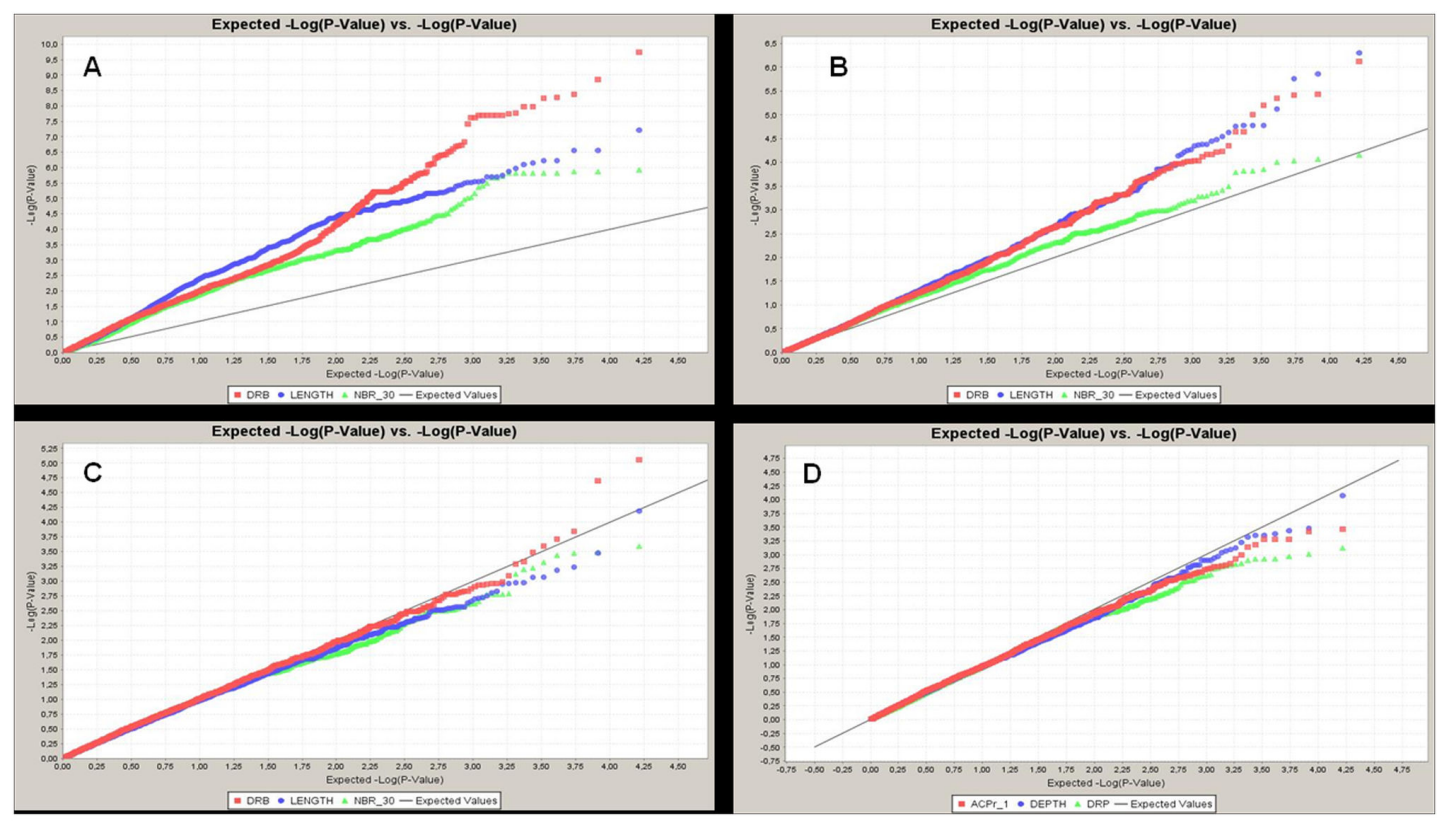
Quantile-quantile plots for four models for three selected traits. A. Model without correction. B. GLM (correction for population structure). C. MLM1 (correction for kinship). D. MLM2 (correction for both population structure and kinship). DRB = deep root biomass (red); LENGTH = maximum root length (blue); NBR_30 = number of roots below 30 cm (green).

**Figure 6:**
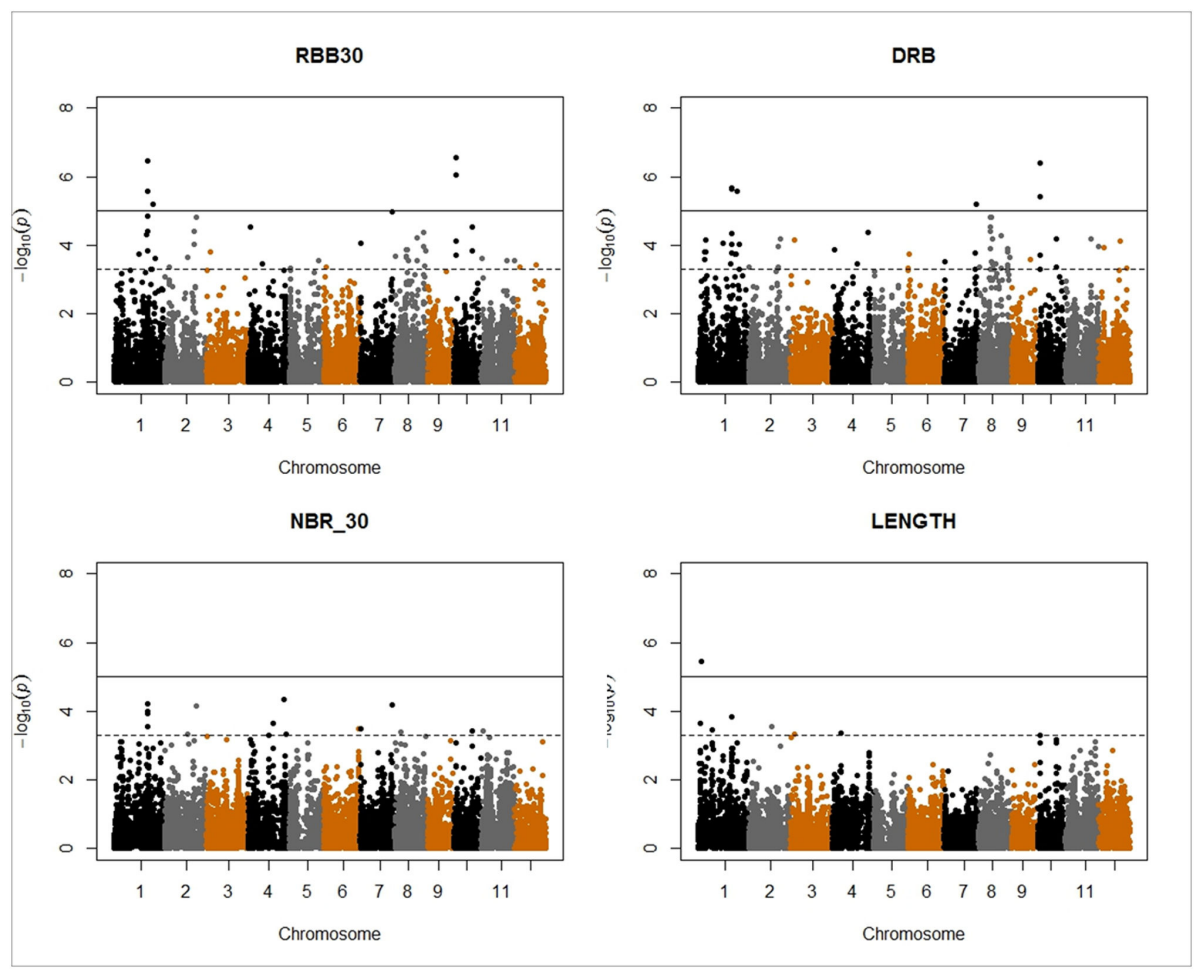
Manhattan plots for four selected root traits. The negative log_10_-transformed *p-values* of each test are plotted against the marker position in the genome. Full line: P=1e-05; dotted line: P=5e-04. RBB30 = root mass below 30 cm; DRB = deep root biomass; NBR_30 = number of roots below 30 cm; LENGTH = maximum root length; .

Most phenotypic traits were affected by panel structure in similar ways. The comparison of the BICs of the three models (GLM, MLM1 and MLM2) showed that MLM2, which included both the population structure and kinship matrix, was the best model for almost all traits (Table 5). MLM1, which included only the kinship matrix, was the best model for NBT, PB and SB. GLM, which included only population structure, was always inferior to the two other models. The smaller number of false positives in MLM compared to GLM is illustrated by the cumulative distribution of *p-values* compared to the uniform distribution, as shown on the quantile-quantile plots for DRB, LENGTH and NBR_30 (Figure 5). The synthetic results of the association mapping run with the best model for each trait are presented in Table 6. The Manhattan plots for four selected root traits (RBB30, DRB, NBR_30 and LENGTH) are presented in Figure 6. Nineteen markers were significantly associated with a trait at P<1e-05, which corresponded to a *q-value* below 0.05; 78 markers were significantly associated with a trait at P<1e-04, which corresponded to a *q-value* below 0.05 in 30 cases (38%) and to a *q-value* between 0.05 and 0.10 in 28 cases (36%), with the remaining 20 markers having *q-values* above 0.10. In a few cases, several markers belonging to the same chromosome segment in full LD were found to have the exact same level of significance (e.g., SNPs in the interval from 34,890,451-34,939,105 bp on chromosome 1 for a range of traits). These segments were less than 50 kb in length, except for one interval on chromosome 8 (460 kb). The number of significant markers at P<1e-04 varied among traits, from 0 to 17. DRB and RBB30, the two traits showing the largest range of phenotypic variation, and NBT were associated with the highest number of significant markers, whereas no significant associations were detected for RB or DRP. Some markers were significantly associated with several traits, which meant that only 51 different sites or segments were found to be significant at P<1e-04 for one of the traits. Among those 51 loci, 53% had a minor allele frequency of less than 10%, which corresponded to the representation of markers with low minor allele frequency in the marker set. Two groups of traits had a high level of co-localization of the significant loci. The first group was composed of traits describing root depth (DRP, RBB30 and NBR_30), with 15 loci significant for two traits (on chromosomes 1, 2, 3, 4, 7, 8, 10, 11 and 12) and five loci significant for all three traits (on chromosomes 1, 2, 7 and 10) among the 24 loci with significance for any of the three traits. RB2030, LENGTH, DEPTH and ACP1 were also related to this first group. The second group of traits was composed of SB, PB and RB0020; among the six loci significant for any of the traits (on chromosomes 4, 5, 7 and 11), four were significant for all three traits. NBT was associated with this group, as was RB, but this association was less clear because the levels of significance were lower for this trait. R_S co-localized erratically. One trait, LLGTH, was almost independent, and another trait, ANGLE, was fully independent of the other traits.

**Table 5:** BIC-based comparison of the three false positive rate control models.

Trait	GLM		MLM1		MLM2	
	-2 ln(L)	BIC	-2 ln(L)	BIC	-2 ln(L)	BIC
LLGTH	1069.0	1104.8	1065.0	1080.4	1026.1	**1067.1**
NBT	404.6	440.5	402.0	**417.4**	378.6	419.5
SB	3.3	39.1	-1.6	**13.8**	-12.2	28.8
LENGTH	1018.6	1054.4	1004.5	1019.8	964.7	**1005.7**
DEPTH	1009.6	1045.5	995.3	1010.7	956.9	**997.8**
ANGLE	1218.0	1253.8	1182.0	1197.4	1136.0	**1177.0**
NBR_30	766.8	802.6	767.1	782.5	733.0	**773.9**
RB0020	-655.8	-620.0	-678.9	-663.5	-666.0	**-619.9**
RB2030	-1092.4	-1056.6	-1090.2	-1074.9	-1069.1	**-1028.1**
RBB30	-1228.4	-1192.6	-1216.0	-1200.7	-1189.2	**-1148.3**
DRB	-931.1	-895.2	-926.4	-911.1	-910.6	**-869.7**
DRP	1058.2	1094.1	1047.5	1062.8	1001.3	**1042.2**
RB	-556.3	-520.4	-567.5	-552.2	-556.9	**-515.9**
PB	57.4	93.2	50.8	**66.1**	39.6	80.5
R_S	-795.7	-759.9	-795.3	-780.0	-783.0	**-742.0**
PCA1	711.5	747.3	708.0	723.4	675.3	**716.3**

GLM = model including population structure only; MLM1= model including kinship only; MLM2 = model including population structure and kinship. L, maximized value of the likelihood function for the estimate model.

The model with the lowest BIC (bold) is considered the best choice for that trait.

**Table 6:** Significant associations detected in the japonica panel (167 accessions) for the measured traits.

QTL	Chr	Position		LLGTH	NBT	SB	RB	PB	ANGLE	RB0020	RB2030	RBB30	DRB	NBR_30	LENGTH	DEPTH	DRP	R_S	PCA1
q1	1	937 008		ns	6.31E-05	ns	ns	ns	ns	ns	ns	ns	ns	ns	ns	ns	ns	ns	ns
q2	1	1 492 914	1 496 524	ns	ns	ns	ns	ns	ns	ns	ns	ns	ns	ns	ns	*3.03E-04*	ns	7.97E-05	ns
q3	1	2 460 843		ns	ns	ns	ns	ns	ns	ns	ns	ns	ns	ns	3.48E-06	ns	ns	ns	1.46E-04
q4	1	5 944 759		ns	ns	ns	ns	ns	ns	ns	ns	ns	6.95E-05	ns	ns	ns	ns	ns	ns
q5	1	21 203 185		ns	ns	ns	ns	ns	ns	ns	ns	*1.75E-04*	8.86E-05	ns	ns	ns	ns	ns	ns
q6	1	28 624 318		ns	ns	ns	ns	ns	ns	ns	ns	3.50E-07	2.15E-06	5.90E-05	*1.42E-04*	6.99E-05	ns	ns	5.58E-05
q7	1	33 364 795		ns	ns	ns	ns	ns	ns	ns	*1.35E-04*	6.37E-06	2.71E-06	ns	ns	1.10E-05	ns	ns	*1.04E-04*
q8	1	34 890 451	34 939 105	ns	1.16E-07	ns	ns	ns	ns	ns	ns	*2.36E-04*	9.30E-05	ns	ns	ns	*4.30E-04*	*4.27E-04*	ns
q9	1	40 960 461		ns	ns	ns	ns	ns	4.33E-05	ns	ns	ns	ns	ns	ns	ns	ns	ns	ns
q10	1	42 706 257		ns	ns	ns	ns	ns	ns	ns	ns	ns	ns	ns	ns	ns	ns	8.12E-05	ns
q11	2	25 228 346		ns	ns	ns	ns	ns	ns	ns	ns	4.05E-05	*1.07E-04*	ns	ns	ns	ns	ns	ns
q12	2	27 406 953		ns	ns	ns	ns	ns	ns	ns	ns	1.53E-05	6.46E-05	6.81E-05	ns	8.09E-05	ns	ns	*2.02E-04*
q13	3	1 248 074		ns	ns	ns	ns	ns	ns	ns	ns	ns	ns	ns	ns	ns	ns	4.90E-05	ns
q14	3	4 103 522	4 137 019	ns	ns	ns	ns	ns	ns	ns	ns	*1.53E-04*	7.25E-05	ns	ns	ns	ns	ns	ns
q15	3	28 355 049		ns	ns	ns	ns	ns	7.01E-05	ns	ns	ns	ns	ns	ns	ns	ns	ns	ns
q16	3	35 694 641		ns	ns	ns	ns	ns	4.33E-06	ns	ns	ns	ns	ns	ns	ns	ns	ns	ns
q17	4	1 708 585		4.09E-06	ns	ns	ns	ns	ns	ns	ns	ns	ns	ns	ns	ns	ns	ns	ns
q18	4	1 913 824		ns	ns	ns	ns	ns	ns	ns	ns	3.00E-05	*1.38E-04*	ns	ns	ns	ns	ns	ns
q19	4	2 913 501	3 411 429	ns	ns	ns	ns	ns	1.38E-05	ns	ns	ns	ns	ns	ns	ns	ns	ns	ns
q20	4	17 514 568		ns	1.48E-05	5.75E-06	*1.11E-04*	8.19E-06	ns	4.45E-05	ns	ns	ns	ns	ns	ns	ns	ns	ns
q21	4	17 604 002		ns	2.93E-06	ns	ns	ns	ns	ns	ns	ns	ns	ns	ns	ns	ns	ns	ns
q22	4	21 386 068		6.67E-07	ns	ns	ns	ns	ns	ns	ns	ns	*3.43E-04*	*2.32E-04*	ns	ns	ns	ns	ns
q23	4	30 477 063		ns	ns	ns	ns	ns	ns	ns	8.47E-05	ns	4.08E-05	4.69E-05	ns	ns	*3.42E-04*	ns	*3.13E-04*
q24	5	4 482 655		ns	ns	ns	ns	ns	4.00E-06	ns	ns	ns	ns	ns	ns	ns	ns	ns	ns
q25	5	15 528 742		ns	1.69E-05	3.78E-05	*1.98E-04*	4.47E-05	ns	7.89E-05	ns	ns	ns	ns	ns	ns	ns	ns	ns
q26	6	20 492 713		5.07E-05	ns	ns	ns	ns	ns	ns	ns	ns	ns	ns	ns	ns	ns	ns	ns
q27	7	1 013 062		ns	ns	ns	ns	ns	ns	ns	ns	9.00E-05	*3.11E-04*	*3.14E-04*	ns	ns	ns	ns	ns
q28	7	10 820 565		ns	ns	4.83E-05	ns	9.21E-05	ns	*4.43E-04*	ns	ns	ns	ns	ns	ns	ns	ns	ns
q29	7	13 278 293		ns	ns	ns	ns	ns	1.42E-05	ns	ns	ns	ns	ns	ns	ns	ns	ns	ns
q30	7	20 921 433		6.54E-05	ns	ns	ns	ns	ns	ns	ns	ns	ns	ns	ns	ns	ns	ns	ns
q31	7	21 509 547		ns	3.08E-05	ns	ns	ns	ns	ns	ns	ns	ns	ns	ns	ns	ns	ns	ns
q32	7	26 566 561		4.77E-05	ns	ns	ns	ns	ns	ns	ns	ns	ns	ns	ns	ns	ns	ns	ns
q33	7	27 436 607		ns	ns	ns	ns	ns	ns	ns	ns	1.02E-05	6.21E-06	6.39E-05	ns	ns	ns	ns	ns
q34	8	2 786 790		4.79E-05	ns	ns	ns	ns	ns	ns	ns	ns	ns	ns	ns	ns	ns	ns	ns
q35	8	9 772 449		ns	ns	ns	ns	ns	ns	ns	ns	ns	2.96E-05	ns	ns	ns	*3.31E-04*	ns	ns
q36	8	10 394 725	10 857 858	ns	ns	ns	ns	ns	ns	ns	*1.21E-04*	*1.34E-04*	1.47E-05	ns	ns	ns	ns	3.02E-04	ns
q37	8	11 754 971		ns	ns	ns	ns	ns	ns	ns	ns	*2.76E-04*	6.40E-05	ns	ns	ns	ns	ns	ns
q38	8	19 669 052		ns	ns	ns	ns	ns	ns	ns	*4.47E-04*	6.24E-05	5.10E-05	ns	ns	ns	ns	ns	ns
q39	8	24 667 998		ns	ns	ns	ns	ns	ns	ns	ns	4.36E-05	ns	ns	ns	*3.81E-04*	ns	ns	ns
q40	8	24 928 700	24 942 846	ns	ns	ns	ns	ns	ns	ns	6.22E-05	ns	*1.27E-04*	ns	ns	ns	ns	ns	ns
q41	9	739 074		ns	ns	ns	ns	ns	ns	ns	ns	ns	*ns*	ns	ns	ns	ns	5.24E-06	ns
q42	10	2 242 059		ns	ns	ns	*4.56E-04*	ns	ns	ns	6.78E-06	2.86E-07	4.09E-07	ns	*4.94E-04*	*1.70E-04*	ns	ns	1.37E-05
q43	10	2 618 906		ns	6.66E-05	*4.46E-04*	ns	ns	ns	ns	ns	ns	ns	ns	ns	ns	ns	ns	ns
q44	10	15 548 603		ns	ns	ns	ns	ns	ns	ns	ns	2.93E-05	6.42E-05	*3.81E-04*	ns	ns	ns	ns	ns
q45	11	22 356 921		ns	ns	ns	ns	ns	ns	ns	ns	*2.87E-04*	6.60E-05	ns	ns	ns	ns	ns	ns
q46	11	23 557 075		ns	6.33E-06	ns	ns	ns	ns	ns	ns	ns	ns	ns	ns	ns	ns	ns	ns
q47	11	24 390 571		ns	5.53E-05	ns	ns	ns	ns	ns	ns	ns	ns	ns	ns	ns	ns	ns	ns
q48	11	28 625 319	28 700 561	ns	ns	4.92E-05	ns	7.57E-05	ns	*3.00E-04*	ns	ns	ns	ns	ns	ns	ns	*3.71E-04*	ns
q49	12	4 885 489		ns	ns	ns	ns	ns	ns	ns	9.27E-05	*4.17E-04*	*1.15E-04*	ns	ns	ns	ns	ns	ns
q50	12	13 430 242		ns	ns	9.61E-05	ns	*1.37E-04*	ns	ns	ns	ns	ns	ns	ns	ns	ns	ns	ns
q51	12	18 332 837		ns	ns	ns	ns	ns	ns	ns	*1.99E-04*	*3.65E-04*	7.60E-05	ns	ns	ns	ns	ns	ns

Position: Polymorphism positions are given with reference to the Os-Nipponbare-Reference-IRGSP-1.0 pseudomolecule assembly [34] An interval is given when several markers in LD have the same significance.

For each QTL, when a test of association with any trait is significant at P<1.0e-05, the *pvalue* of the tests of association with other traits (up to P<5e-04) is given in italics.

### Localization of significant loci

**Figure 7:**
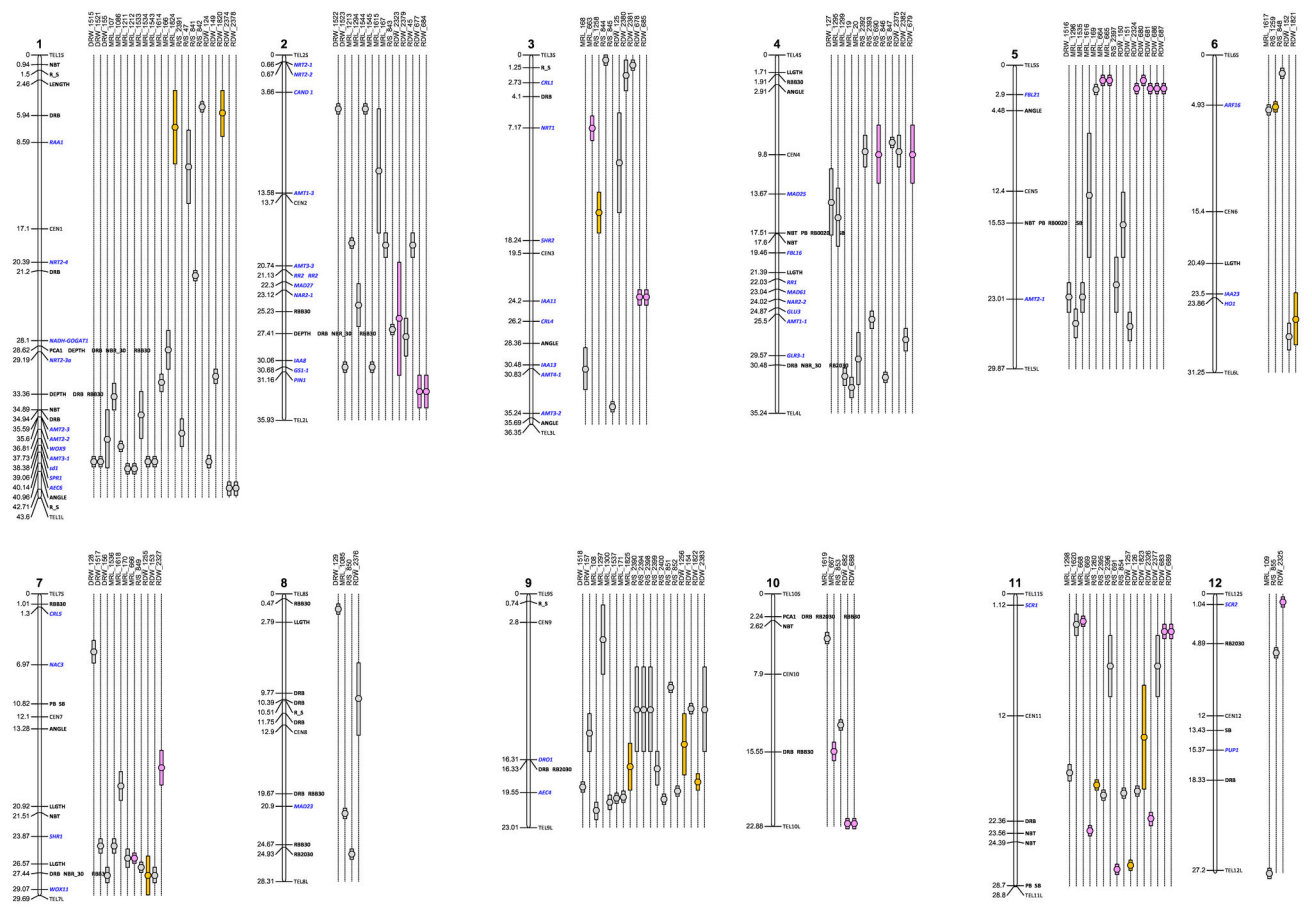
Relative positions of significant markers, genes and QTLs for corresponding root traits. Significant markers are in black and genes in blue on the chromosome bodies .QTLs in orange and pink were detected in japonica x japonica and indica x indica mapping populations respectively. QTLs in grey were detected in other population types (indica x japonica, japonica x indica or japonica x aus). MRL = Maximum root length DRB= deep root biomass; RB = root biomass; R_S =root to shoot ratio. Data were extracted from the rice module from TropgeneDB for the root genes ("EURoot genes" set) and the QTLs (http://tropgenedb.cirad.fr/tropgene/JSP/interface.jsp?module=RICE). The QTL numbers correspond to their ID in this database.

Among the 51 loci significantly associated with one or more traits, 19 were in predicted genes, and ten of these encoded expressed or hypothetical proteins without known functions. The 32 other loci were in intergenic regions. Among the 37 loci associated with a root trait (excluding loci associated only with LLGTH, NBT, SB or PB), 20 co-localized with root QTLs on chromosomes 1, 2, 3, 4, 5, 7, 8, 9, 10 and 11 (Figure 7). The 17 remaining loci did not co-localize with any QTLs considered in this study. When focusing on the 12 QTLs detected only in japonica x japonica mapping populations, i.e. in the same genetic background than the association panel, 4 loci on chromosomes 1, 7 and 9 co-localized with those QTLs. There was almost no co-localization with rice genes with demonstrated role in root development. No marker co-localized with *sd1*, the major semi-dwarfism-inducing gene located on chromosome 1, which is known to influence plant biomass. In fact, the semi-dwarfism allele, which is very common in improved irrigated varieties, is not commonly used in upland rice breeding. Close physical proximity (20 kb) was observed between a marker associated with both DRB and RB2030 and *Dro1*, a cloned root angle QTL located on chromosome 9, but the significance of the marker was slightly below the threshold of 1e-04 (Table S5). Given the level of LD in the panel (r20.6 at a distance between markers below 20 kb), we also surveyed the genes that were in an interval of +/-25 kb on both sides of the significant markers. We found 521 genes, of which 261 had a predicted function (Table S6). Among these 261 genes, kinases (27) were over-represented relative to their overall presence in the rice genome (10.3% versus 3.5%). Several other genes appeared as potentially relevant candidates: multicopper oxidases (three in a cluster on chromosome 1); gibberellin dioxygenases (five on chromosomes 1, 2 and 11); glutathione-S transferases (two on chromosomes 1 and 11); and elongation factors (five).

## Discussion

We performed an association mapping study for root traits in the rice japonica group using a medium-throughput hydroponic root phenotyping system with glass beads and marker data obtained by GBS.

Hydroponics enables an easy observation of the root system but does not permit to assess root growth reaction in response to drought. However, in rice, what is crucially important for productivity under drought stress situations is the presence of deep roots prior to the onset of stress [3,56]. Under severe drought stress, root plasticity is limited, with no increase in root distribution in depth, to the contrary of what is occurring in maize [57]. Therefore the constitutive expression of the genetic potential in the absence of stress, which is what is assessed under hydroponic conditions, is seen as an important element by rice breeders [58]. The selection of varieties introgressed with a QTL for root depth detected under favorable conditions has already led to the release of a variety drought resistant under rainfed conditions [17]

The hydroponic system with glass beads has the advantage over hydroponics without substrate or homogeneous media such as agar, to involve a granular substrate and to impose a physical constraint to root growth through glass beads. This system is therefore expected to be closer to field conditions, in which the soil strength increases when the soil dries, although a formal comparison remains to be done. The effect of mechanical impedance on roots has been investigated [59,60]. Mechanical impedance decreases elongation rate, increases root diameter and modifies branching but, because compensation occurs, does not affect total root biomass. We chose to grow plants to an age of 30 days to maximize the differences among accessions, but this choice, imposing the use of large rhizoboxes, reduced the throughput of the experiment. The number of accessions that can be phenotyped in the system is limited by the trade-off between the need to create conditions under which plants express relevant variation and to accurately control the sources of environmental phenotypic variation, and the requirement of association mapping in terms of panel size. Using simulations, Kang et al. [51] demonstrated a dramatic increase in power by using replicate measurements in association mapping. However, even with only two replicates, the trait heritabilities we obtained through our experimental design were generally high. 

The issue of whether any phenotyping system under controlled conditions can accurately represent what occurs in the field is always a subject of debate. The development of the plant root system in the Rhizoscope is reasonably well correlated with what has been observed in plants grown in soil columns for several traits [29] for the 30 accessions shared between the two studies, although the resource conditions and plant age were not identical in the two systems. The transferability from the Rhizoscope to field situations remains to be evaluated. In a field, the plant genetic makeup interacts with multiple physical, chemical and biological soil factors, often heterogeneously distributed, and genotype x environment interactions will likely lead to differences in root system growth and architecture. The simplification permitted by near optimal controlled conditions is useful when the objective is to assess the genetic potential on a comparative basis for a set of accessions. However, as demonstrated by Rich and Watt [61], a better understanding of how soil conditions and inter-plant competition for space and resources regulate root architecture is needed to translate this potential into information relevant to different field conditions.

Root spread angle has been proposed as a proxy for root depth for some cereal species such as rice [62], and sorghum [63] because the angle is easier to measure and show a good heritability, although the relationship does not hold true for other species such as durum wheat [64]. The relationship between narrow root growth angle relative to the vertical and root depth, indirectly observed by Kato et al. [62] using the basket phenotyping system, was not observed in our system. This lack of relationship was observed not only in the present japonica panel but also in an indica panel (Audebert et al., unpublished data) and therefore cannot be attributed to the genetic background. This discrepancy may result in part from the fact that the two studies do not measure exactly the same variable. The basket method computes the average frequency of roots above a given angle, assessing the whole root system in three dimensions, whereas our system only counts the most external roots in a system that is two-dimensional. Another possible explanation for the differing results is that the effect of gravimetric forces is partly compensated by Archimedes's push under hydroponic conditions. 

The phenotyping of the panel showed results that were fully consistent with previous observations in terms of characteristics of the sub-populations [5]. The sub-population root characteristics matched their different adaptations: subpopulations 1, 2 and 3, with deep roots, are composed of varieties adapted to tropical or equatorial aerobic upland systems while subpopulations 4 and 5, with shallow roots, are composed of accessions adapted to the anaerobic temperate irrigated and tropical rainfed lowland systems respectively. Accessions from subpopulation 6, with their small above ground and below ground biomasses are adapted to high input aerobic situations with high plant density only common in Latin America [58].

The GBS method yielded a large number of markers. Their distribution was not completely even across the genome but was sufficiently homogeneous to let only few and small loosely covered genomic regions. A few segments with low marker density (e.g., segments on chromosomes 4 and 7) correspond to zones that have previously been identified as SNP deserts in the species [65], but most seem to be specific to the japonica group. Huang et al. [20], working with a panel including indica and japonica accessions, found that approximately 10% of the SNPs were nearly fixed (frequency >95% in one sub-species and <5% in the other), and 3.5% were completely fixed. The risk of encountering an important proportion of markers with very low to low minor allele frequency was expected to be high in a panel belonging to one sub-species. We made a deliberate choice to focus on the japonica group because the use of a core collection representative of the overall genetic diversity of *O. sativa* carried the alternative risk of having true associations appear as false negatives given the correlation between phenotypic variability and population structure for root traits in rice [5,29]. If the variation in a trait is caused by alleles with low frequency, there is a high risk of not detecting the associations due to a lack of statistical power [24]. Conventional mapping, which ensures a balanced allelic frequency, is better adapted to such situations. The use of a larger population size is another way to limit the problems of variance heterogeneity between highly unbalanced genotypic classes. However, depending on the phenotyping system, very large population sizes are often detrimental to phenotypic precision, or are simply unaffordable.

The LD decay we observed (150 kb) is in the range of those reported by Mather et al.[23], Huang et al. [22], and Xu et al. [30] in japonica backgrounds, which vary between 150 and 180 kb, although our panel involves a relatively large proportion of breeding lines. This relatively slow LD decay limits the resolution of association mapping, but the 16,444 markers genotyped provide sufficiently high genome coverage to ensure that most genes are in LD with one or more markers. 

We detected several markers that are significantly associated with root traits, showing that genome-wide association mapping can be used to dissect those traits in a tropical japonica panel. As shown in Table 6, a few markers were found to be associated with several traits that are linked by construction or because of pleiotropy, i.e., traits determining biomass on the one hand, and traits determining root depth on the other hand. These two groups of traits were also the ones showing the highest within group phenotypic correlations.

The comparison of the positions of the markers detected with previously published data showed that some of the identified markers co-localized with QTLs, but almost none co-localized with any of the limited number of genes presently known to influence root growth in rice. A certain proportion of these co-localizations might be due to chance because the sum of the confidence intervals of the 137 QTLs covered 52 % of the genome. However, 46 % of the markers that were significantly associated with root traits did not colocalize with any known QTL. This finding was expected because the large majority of the root QTL studies focused on indica x japonica mapping populations [14]. Only 4 studies (coresponding to 7% of the detected root QTLs) used japonica x japonica mapping populations and none a tropical japonica x tropical japonica mapping population. By focusing on the within-japonica diversity, which has only rarely been investigated, one of our objectives was to identify new loci involved in root development. This seems to be the case. Conversely, some highly supported meta-QTLs, such as mMRL_9-2 [14], did not co-localize with any significant markers detected here. The allele at this meta-QTL may be fixed in the japonica sub-species, as could be suspected from the low polymorphism observed around its position in the japonica panel. 

Because the LD in this panel spans long distances and because the marker density is 22.5 kb on average, the resolution of association mapping is much broader than the single-gene level. An analysis of the genes near the significant markers suggested several possible candidate genes based on data from *Arabidopsis* or other evidence. For instance, the multicopper oxidase domain-containing genes are known to play a role in root development in low phosphate situations [66], glutathione-S-transferases have been reported to be involved in meristem maintenance and the growth of lateral roots[67], and several LRR-LRKs have been found to be associated with a root mutant phenotype in rice (Dievart, personal communication). However, further evidence is needed to demonstrate that these genes are indeed involved in root growth in rice.

Although several significant markers were detected, we found fewer markers than we anticipated given that linkage mapping studies conducted in smaller biparental populations detected many more QTLs per trait [14]. The limited number of highly significant associations may be partly attributed to the fixation of some QTLs in the japonica panel, as noted above, but the number of markers may also be partly responsible. Although theoretically more than sufficient, considering the panel average LD, the marker density may be too low in zones where LD decays more rapidly or breaks down due to recombination events. Most functional polymorphisms are probably absent from our marker set. As demonstrated by Segura et al. [68], when LD is not at its maximum, the power of the association study decreases sharply when the functional variants are untyped. A high-quality 950,000 SNP array is under development for rice [31], and our panel will be genotyped with this chip, enabling more powerful analyses in the future. In addition, new methods might be used to increase the detection power. As suggested by Koerte et al. [69], correlated traits essentially represent a form of replication. The joint analysis of correlated traits might provide additional power in detecting associations, as shown by the same team through simulations. In the same way, multi-locus mixed models, similar to the composite interval mapping used in classical linkage mapping, may be helpful in situations involving loci with moderate to large effects [6].

This association mapping study was conducted in a japonica panel. We intend to conduct a similar study in an indica panel of similar size to determine whether the associations detected are specific to the japonica sub-species or are common to both sub-species. A pooled analysis of the two panels might provide additional detection power as a result of the larger population size, at least for markers that are not correlated with population structure.

## Supporting Information

Figure S1 **Distribution of marker allelic frequency along the genome.**
(PDF)

Figure S2 **NJ tree with the six different sub-populations detected by Structure shown in different colors; admixed accessions are shown in black.**
(PDF)

Table S1 **List of the accessions included in the panel with their country of origin and Structure group (xlsx).**
(XLSX)

Table S2 **Composition of the nutrient solution.**
(XLSX)

Table S3 **Average linkage disequilibrium between marker pairs according to chromosomes and distance between markers.**
(XLSX)

Table S4 **Phenotypic correlation between traits.** P-values are indicated in smaller font below the correlation values.
(XLSX)

Table S5 **Detailed results of the association mapping tests.**
(XLSX)

Table S6 **List of annotated genes located around the significant markers (+/- 25 kb).** The positions of the QTLs detected in this study are shown in orange.
(XLSX)
