# Supplementary figures and images for: Genome-Wide Association Mapping of Root Traits in a Japonica Rice Panel
Source: PLoS One. 2013 Nov 5;8(11):e78037. doi: 10.1371/journal.pone.0078037 (PMC3818351; doi:10.1371/journal.pone.0078037)

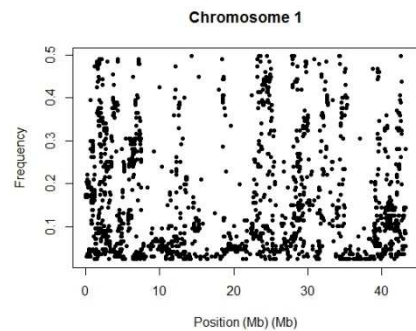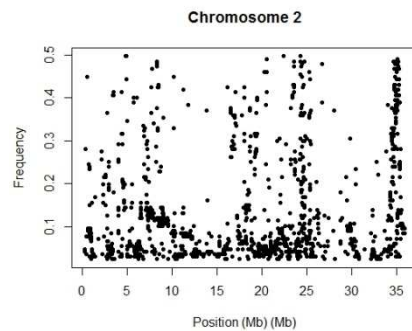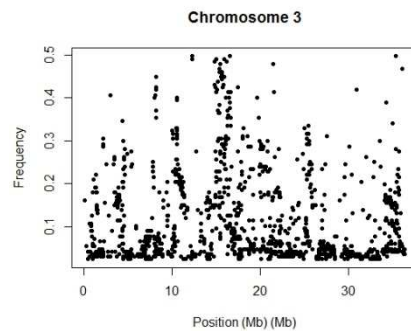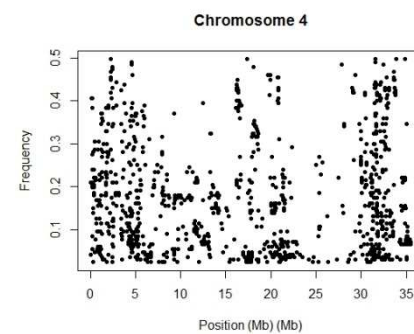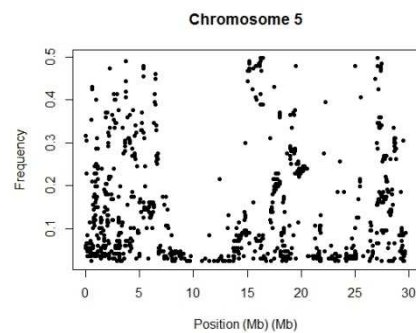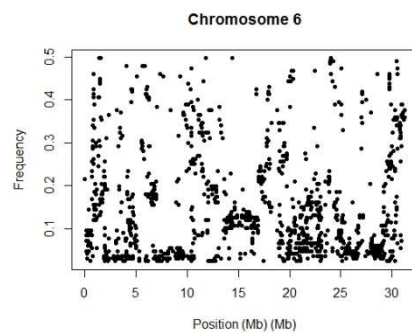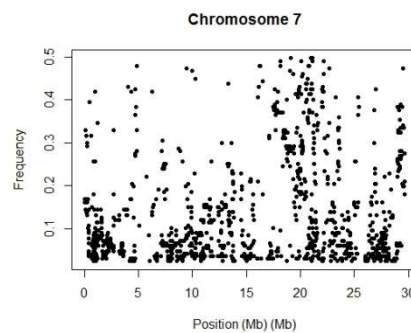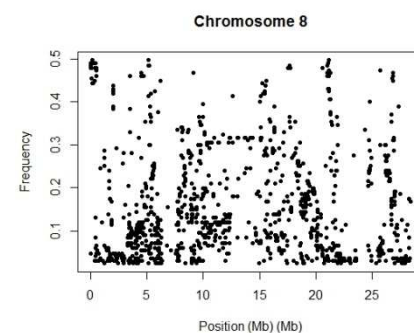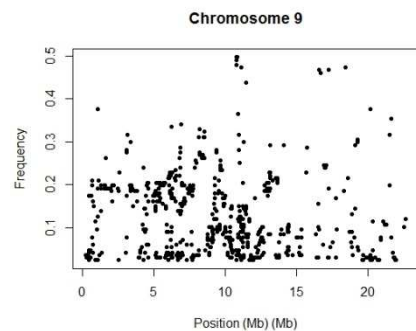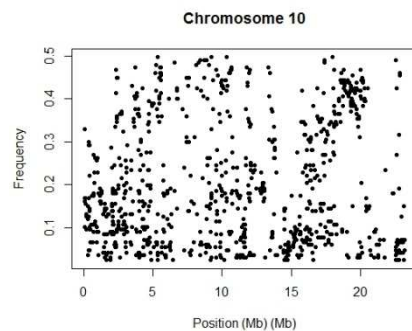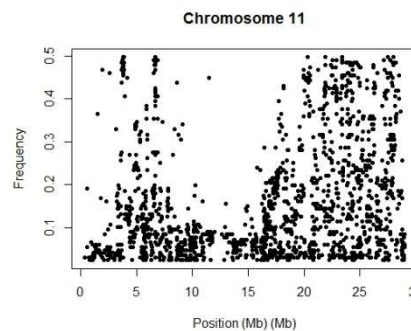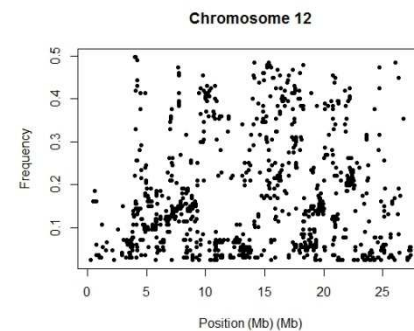

Supplement: Figure S1 — Distribution of marker allelic frequency along the genome. (PDF) [file pone.0078037.s001.pdf]

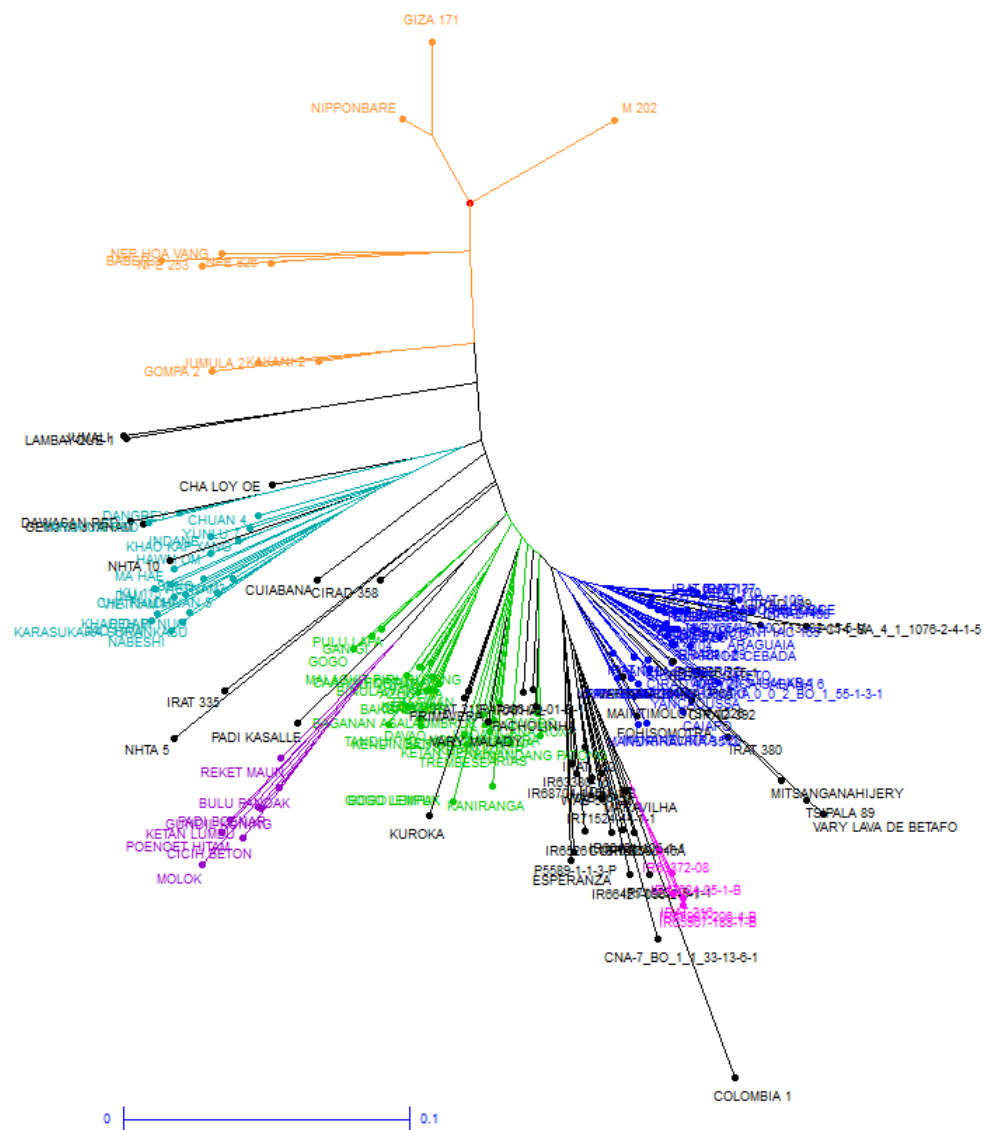

Supplement: Figure S2 — NJ tree with the six different sub-populations detected by Structure shown in different colors; admixed accessions are shown in black. (PDF) [file pone.0078037.s002.pdf]
